# Association of State Legislation Restricting Reproductive and LGBT Rights With Infectious Diseases Fellowship Match Rates

**DOI:** 10.1093/ofid/ofaf534

**Published:** 2025-09-23

**Authors:** Michael Z Chen, Aniruddha Hazra, Alan L Hutchison, Anna E Czapar

**Affiliations:** Department of Internal Medicine, University of Chicago Medical Center, Chicago, Illinois, USA; Section of Infectious Diseases and Global Health, University of Chicago Medical Center, Chicago, Illinois, USA; Section of Gastroenterology, Hepatology, and Nutrition, University of Chicago Medical Center, Chicago, Illinois, USA; Department of Infectious Disease, Loyola Medicine, Maywood, Illinois, USA

**Keywords:** fellowship, LGBT rights, match rates, medical education, reproductive rights

## Abstract

**Background:**

Infectious diseases (ID) fellowship match rates have declined over the last decade. Previous studies have identified factors that influence interest in ID and fellowship match rates, but the association of state legislation restricting reproductive and/or lesbian, gay, bisexual, and transgender (LGBT) rights with ID fellowship match rates is unknown.

**Methods:**

All adult ID fellowship programs in the United States from 2017 to 2025 were categorized as located in states, districts, or territories with or without restrictions on reproductive and/or lesbian, gay, bisexual, and transgender rights. Programs were also categorized by academic status as academic, university-affiliated community, or pure community programs. Match rates were compared between programs when stratifying by state restrictions, academic status, and both status using χ^2^ tests.

**Results:**

In 6 of the 9 years studied, match rates of adult ID fellowship programs were higher to a significant degree for academic programs in permissive states than for those in restrictive states (2017–2018, 2021–2023, 2025; *P* = .002 to *P* = .02). In the same period, there were no differences in match rates between university-affiliated community programs or pure community programs in states with and without restrictions.

**Conclusions:**

The presence of legislation restricting reproductive and/or LGBT rights was associated with significantly reduced match rates in academic programs but not in university-affiliated community programs or pure community programs. Academic fellowship programs represent the majority of ID fellowship programs and must make note of this when recruiting fellows.

## Teaser

Infectious diseases fellowship match rates have declined over the last decade. This article shows that state legislation restricting reproductive and/or LGBT rights is associated with significantly decreased match rates in academic fellowship programs from 2016 to 2025.

## Graphical Abstract

**Graphical Abstract:**
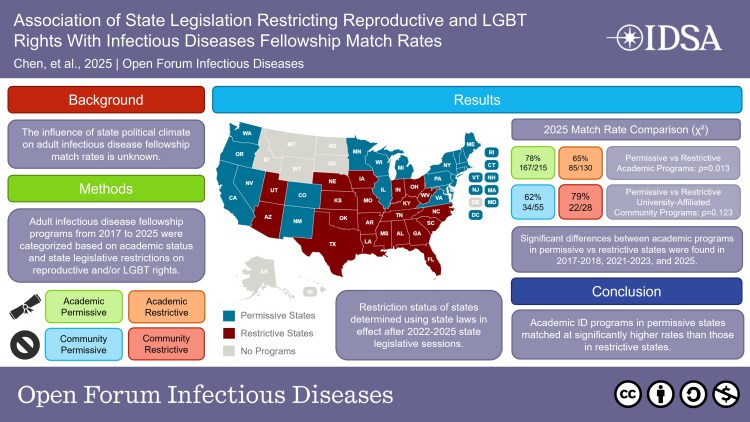

Over the last decade, infectious diseases (ID) fellowship match rates in the United States have declined. In certain years, up to 50% of ID programs have not filled all their open positions through fellowship match process [1]. This decline does not match increasing workforce needs, with nearly 80% of counties in the United States representing 208 million citizens lacking a single practicing ID physician [2]. Where ID physicians choose to practice is linked to the states in which they undergo fellowship training, and state political climates have been rapidly changing over the last decade [3]. State political climate began rapidly changing after 2022 when the US Supreme Court overturned decades of precedent establishing the constitutional right to abortion set in 1973 by *Roe v Wade* through the decision in *Dobbs v Jackson Women’s Health Organization* [4, 5]. In the wake of this decision, many states have enacted legislation restricting reproductive and/or lesbian, gay, bisexual, and transgender (LGBT) rights [6, 7].

New restrictions on reproductive rights have reduced application rates to residency programs in states that enacted new restrictions, particularly in obstetrics and gynecology programs [8, 9]. As of 2021, the average age at first pregnancy among physicians of childbearing potential is 32 years [10]. Such restrictions thus directly affect physicians applying to fellowship programs because these applicants tend to be in their 20s to 30s and may consider becoming pregnant during fellowship training or shortly afterward. These restrictions may also be more salient to ID fellowship program applicants because these applicants identify as women and LGBT more frequently than applicants to all internal medicine subspecialty fellowship programs as a whole [11]. We hypothesize that changing state political climates are associated with changes in ID fellowship match rates because of the diversifying demographics of ID fellowship applicants. This study describes how state legislation enacted after 2022 and aimed at restricting reproductive and/or LGBT rights is associated with ID fellowship match rates.

## METHODS

Every year, the National Resident Matching Program (NRMP) organizes the filling of ID fellowship positions across the United States, the District of Columbia, and Puerto Rico, through the Specialties Matching Service, colloquially known as the NRMP match. The NRMP subsequently publishes a yearly list of all programs, listing the number of open positions in each program and the number of these positions directly filled through the NRMP match [12–15]. Since 2023, the NRMP also publishes the self-reported demographic data of applicants [11]. Starting in the 2017 match, ID fellowship programs across the United States transitioned to an all-in match and all open positions became filled through the NRMP match process.

The demographics of applicants to all ID and all internal medicine subspecialty fellowships from 2023 to 2025 with regard to sexual orientation and gender identity were tabulated as an overall proportion across those 3 years. The proportion of ID fellowship applicants who identified as women was compared with that of applicants to all internal medicine subspecialty fellowships using a 2-proportion *z* test. The same comparison was made between the proportion of ID fellowship applicants who identified as LGBT compared with that of applicants to all internal medicine subspecialty fellowships. Differences were considered significant at *P* < .05 .

All 50 states in the United States, the District of Columbia, and Puerto Rico were classified as with or without reproductive restrictions, using data from the *New York Times* abortion ban database [6]. Although the United States formally designates the District of Columbia as a federal district and Puerto Rico as a territory, this article will also refer to those 2 regions as “states” because both are administered similarly to states with regard to enacting local legislation. If a state enacted new legislation limiting abortion before 20 weeks of gestation through legislative action and executive approval after the overturning of *Roe v Wade* in 2022, that state was classified as having reproductive restrictions. States that never enacted new legislation limiting abortion were classified as not having reproductive restrictions. If a state successfully enacted legislation limiting abortion after 2022 but then overturned it through ballot initiatives or court decisions, it were classified as restrictive for the full period studied. This is because enacting restrictive legislation requires overall aligned political will at the highest levels of a locality's elected government and reflects that government's attitude.

States were similarly classified as with or without LGBT restrictions using data from the Human Rights Campaign’s State Equality Index database [7]. If that Index indicated that a state had negative legislation targeting freedom of LGBT individuals, such as religious refusal of service laws, that state was classified as having LGBT restrictions. States without such restrictive laws or with laws that specifically protect against discrimination of LGBT individuals were classified as not having LGBT restrictions.

States with neither reproductive nor LGBT restrictions were classified as permissive states. States with reproductive restrictions, LGBT restrictions, or both, were classified as restrictive states. Each fellowship program was then classified by academic status based on how it self-identified in the Fellowship and Residency Electronic Interactive Database Access (FREIDA) system [16]. Using these data, programs were classified as academic, university-affiliated community, or pure community programs. Thus, all fellowship programs could be ultimately categorized in 1 of 6 categories based on academic status and restriction status of the states in which they are located (academic, permissive state; academic, restrictive state; university-affiliated community, permissive state; university-affiliated community, restrictive state; pure community, permissive state; or pure community, restrictive state).

The numbers of matched, unmatched, and total positions of every adult ID fellowship program from 2017 to 2025 in each of the 6 categories were tabulated [12–15]. Comparisons between the number of matched and unmatched positions each year between nominal variables were performed using χ^2^ tests (Supplementary Tables 1–7). Differences were considered significant at *P* < .05. Match rates were calculated as the cumulative percentage of successfully filled positions in each program. For example, a 90% match rate for academic permissive programs in a year indicates that 90% of all positions offered by academic permissive programs that year were directly filled by the NRMP match process and 10% remained unfilled. Fellowship match years were defined by the year in which newly matched fellows start fellowship training.

This study did not include any factors necessitating patient consent.

## RESULTS

From 2017 to 2025, the number of ID fellowship positions filled directly through the NRMP match process rose from 392 to 450. There were fellowship programs in 23 permissive states, including the District of Columbia and Puerto Rico (California, Colorado, Connecticut, District of Columbia, Illinois, Maine, Maryland, Massachusetts, Michigan, Minnesota, Nevada, New Hampshire, New Jersey, New Mexico, New York, Oregon, Pennsylvania, Puerto Rico, Rhode Island, Vermont, Virginia, Washington, and Wisconsin). There were fellowship programs in 21 restrictive states (Alabama, Arizona, Arkansas, Florida, Georgia, Indiana, Iowa, Kansa, Kentucky, Louisiana, Mississippi, Missouri, Nebraska, North Carolina, Ohio, Oklahoma, South Carolina, Tennessee, Texas, Utah, and West Virginia). Nearly all states that had either reproductive or LGBT restrictions also had the other. One state each had reproductive restrictions alone (Iowa) or LGBT restrictions alone (Kansas). Eight states had no adult ID fellowship programs (Alaska, Delaware, Hawaii, Idaho, Montana, North Dakota, South Dakota, and Wyoming). Of the 58 new fellowship positions that opened during this period, 23 (40%) were in permissive states and 35 (60%) in restrictive states.

From 2023 to 2025, 51.4% (409 of 795) of ID fellowship applicants identified as women compared with 43.0% (8043 of 18 699) of all internal medicine subspecialty fellowship applicants (*P* < .001). In the same period, 13.6% (108 of 795) of ID fellowship applicants identified as LGBT compared with 4.8% (894 of 18 699) of all internal medicine subspecialty fellowship applicants (*P* < .001).

The overall ID fellowship match rate varied from 2017 to 2025. Overall match rates were stable from 2017 (80% [312 of 392]) to 2020 (79% [322 of 406]) before increasing to 88% (365 of 416) in 2021. Overall match rates decreased from 2022 (82% [365 of 416]) to 2024 (67% [303 of 450]) before improving to 70% (316 of 450) in 2025. Many programs routinely do not fill their open positions through the NRMP match. Of the 179 programs in the 2025 match, 77 (43%) did not fill any of their open positions and had to recruit all of their fellows outside the NRMP match. An additional 11 programs (6%) filled some but not all of their open positions and similarly had to recruit fellows outside the match. As of 2025, approximately 70% of programs identify as academic programs, 20% as university-affiliated community programs, and 10% as pure community programs. In all years, there are more available positions than total applicants.

**Figure 1.**
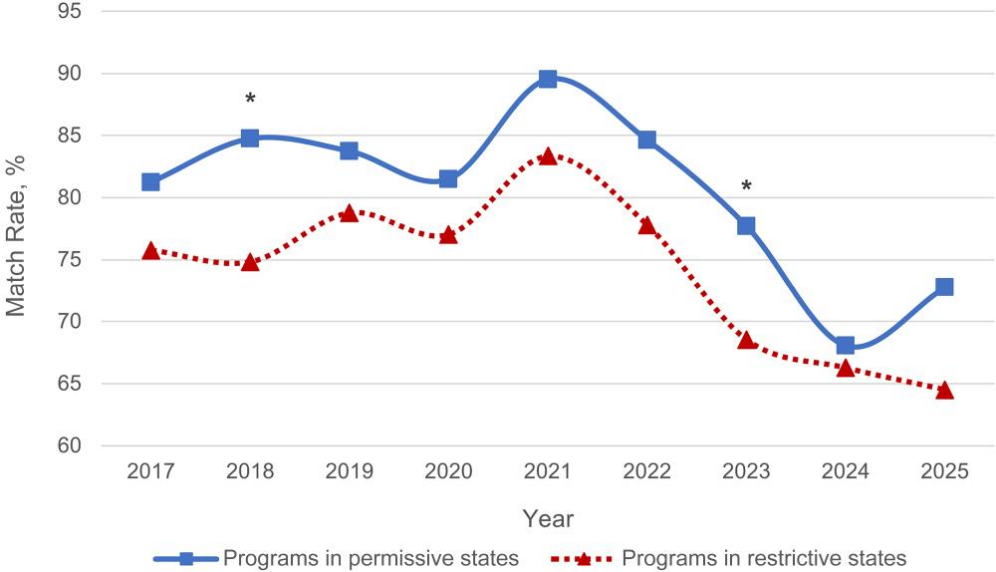
Match rates of fellowship programs by state restriction status from 2017 to 2025. Significant values are indicated by an asterisk: *P < .05.

Match rates were compared between programs located in permissive and restrictive states regardless of academic status. Match rates were significantly higher for programs in permissive states than for those in restrictive states in 2018 (85% vs 75%; *P* = .02) and 2023 (78% vs 69%; *P* = .03) (Figure 1 and Supplementary Table 1). Though not to a significant degree, the match rates of programs in permissive states remained higher than those of programs in restrictive states in all other years from 2017 to 2025.

**Figure 2.**
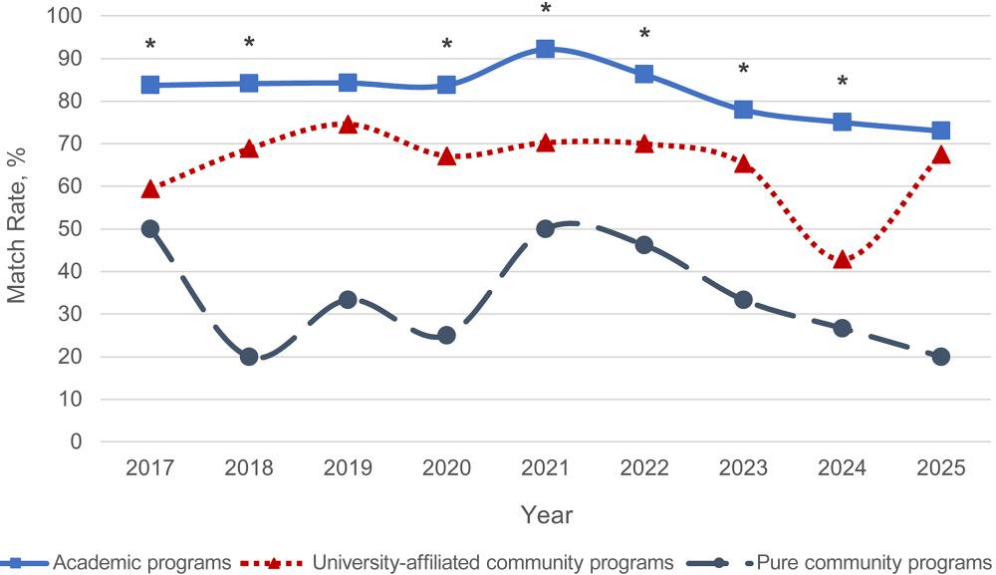
Match rates of all programs by academic status from 2017 to 2025. Significant values comparing match rates between academic and university-affiliated community programs only are indicated by an asterisk: *P < .05.

Match rates were next compared between academic and university-affiliated community programs regardless of state restriction status. Rates were significantly higher in academic programs than in university-affiliated community programs in all years from 2017 to 2025 except for 2019 and 2025 (*P* < .001 to *P* = .02) (Figure 2 and Supplementary Table 2).

**Figure 3.**
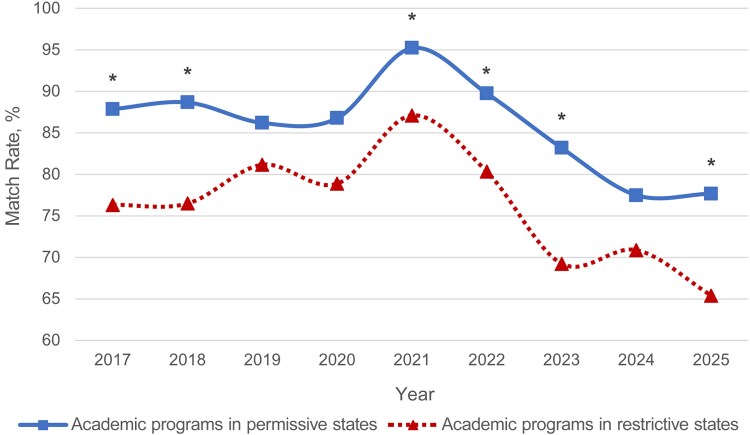
Match rates of academic fellowship programs by state restriction status from 2017 to 2025. Significant values are indicated by an asterisk: *P < .05.

Match rates in academic programs were also compared between academic programs in permissive states and those in restrictive states. Academic programs in permissive states always had higher match rates than those in restrictive states from 2017 to 2025. This was to a significantly higher degree in 2017–2018, 2021–2023, and 2025 (*P* = .002 to *P* = .02) (Figure 3 and Supplementary Table 3).

**Figure 4.**
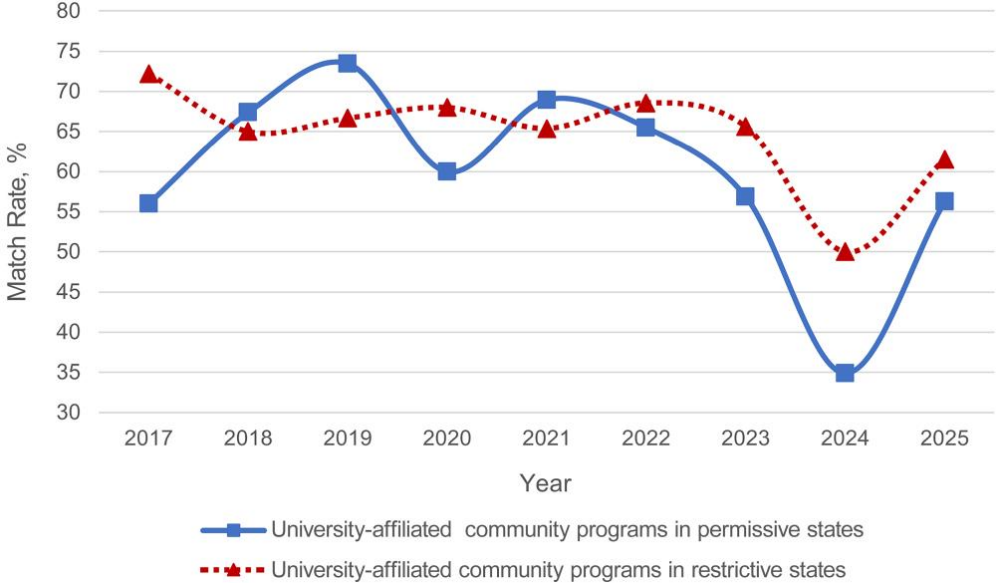
Match rates of university-affiliated community programs by state restriction status from 2017 to 2025. Significant values are indicated by an asterisk: *P < .05.

Match rates in university-affiliated community programs were then compared between those in permissive and those in restrictive states. Match rates in both types of university-affiliated community programs fluctuated from 2017 to 2025, but neither consistently matched at higher rates than the other. There were no years in which university-affiliated community programs in permissive states matched at higher rates than those in restrictive states to a significant degree, and vice versa (Figure 4 and Supplementary Table 4).

**Figure 5.**
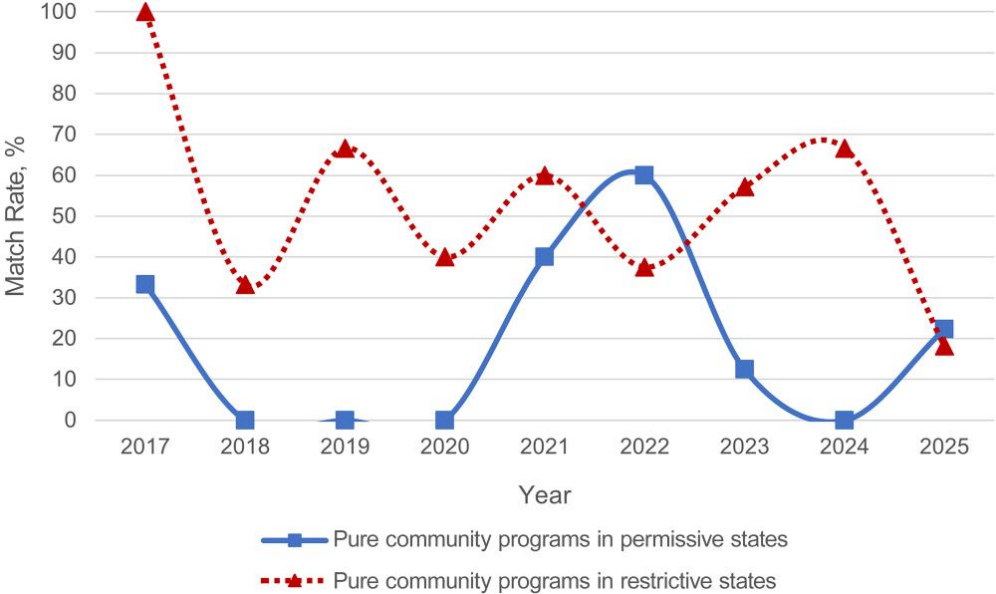
Match rates of pure community programs by local restriction status from 2017 to 2025. Significant values are indicated by an asterisk: *P < .05.

There were no differences in match rates between pure community programs in permissive states and restrictive states from 2017 to 2025 (Figure 5 and Supplementary Table 5). Academic programs matched at significantly higher rates than pure community programs from 2018 to 2025 (*P* <.001 to *P* = .001) (Supplementary Table 6). University-affiliated community programs matched at significantly higher rates than pure community programs in 2018–2020, 2023, and 2025 (*P* < .001 to *P* = .03) (Supplementary Table 7). The sample size of positions offered by pure community programs, however, was small and ranged from 4 to 20, limiting the significance of results involving pure community programs.

## DISCUSSION

The current study evaluated the association between state legislation restricting reproductive and/or LGBT rights and the US adult ID fellowship program match rate from 2017 to 2025. Previous studies have identified factors intrinsic to fellowship programs, such as specialty training tracks and the fellowship program’s social media presence, as positively influencing match rates to specific programs [17]. To our knowledge there have not been any studies evaluating the effect of state political climates on ID fellowship match rates. Our findings demonstrate that fellowship match rates in academic ID fellowship programs tend to be significantly lower in states with restrictive political climates than in those with permissive political climates.

In 2022, most medical students and internal medicine residents had been born after 1973 and only knew a nation in which the constitutional right to abortion had been enshrined through *Roe v Wade* (1973). The decision of *Dobbs v Jackson Women's Health Organization* (2022) disestablished the right to abortion and caused a seismic shift that immediately altered political climate across states. Many states immediately enacted restrictions on abortion and used this momentum to enact restrictions on LGBT individuals if they had not already begun doing so [7].

Restrictions on reproductive and LGBT rights continue to be of significant national discourse. Enacted in 2025, the Tennessee Medical Ethics Defense Act passed allows healthcare providers to legally refuse to provide nonemergent medical care to patients who violate their own personal religious, moral, or ethical beliefs [18]. This law has already been used to refuse prenatal care to an unmarried woman by a provider who objected to pregnancy outside of marriage [19]. This law can also be used to refuse care to LGBT patients if the provider desires. Several restrictive states have also urged the US Supreme Court to overturn other existing rights extended toward LGBT individuals, such as marriage equality, despite the affirmation of this right in *Obergefell v Hodges* (2015) and the *Respect for Marriage Act* (2022) [20].

These restrictions on reproductive and LGBT rights are highly relevant to women of childbearing age, and younger generations of Americans who identify as LGBT who are increasingly represented among medical school matriculants and ID fellowship applicants [21]. As of 2024, a majority of medical school matriculants now identify as women, and nearly 1 in 8 identify as LGBT [22–24]. As we found, applicants to ID fellowships from 2023 to 2025 have a significantly higher level of diversity than applicants to all internal medicine subspecialty fellowships, with regard to gender and sexual orientation. Because there are consistently more open ID fellowship positions than applicants each year, ID fellowship applicants can be highly selective in where they choose to train, especially if they believe that restrictions on reproductive and LGBT rights will directly affect their life experiences during fellowship training.

When analyzing trends in overall match rates from 2017 to 2025, no formal data exist to explain the 2021 bump in match rate to 88% and subsequent decline to 70% in 2025, but there is anecdotal evidence surrounding the coronavirus disease 2019 (COVID-19) pandemic. The 2021 NRMP match occurred shortly after the start of the COVID-19 pandemic when COVID-19 was of incredible research interest and many trainees were taking care of numerous inpatients with COVID-19. The rise in applications to both medical schools and ID fellowships in during this time has been called the “Fauci effect” due to the visible public health efforts of Dr Anthony Fauci [25]. Reasons for declining match rates from the 2022 NRMP match onward are multifactorial. These include continued reduced physician compensation by Medicare, declining public trust in public health institutions such as the US Centers for Disease Control and Prevention, and assaults on the same public health institutions by elected state and federal officials [25–27].

When ID fellowship programs were stratified by permissive and restrictive states, match rates did not differ consistently. Only in 2018 and 2023 did programs in permissive states match at significantly higher rates than programs in restrictive states. However, controlling for academic status revealed a sharp divergence between programs in permissive and restrictive states. In the subset of academic fellowship programs, match rates were significantly higher for academic programs in permissive states than for those in restrictive states in 6 of the 9 years studied. This difference was present in 2017–2018, 2021–2023, and 2025 but not in 2019–2020 or 2024. In contrast, there were no significant differences in match rates between in permissive and restrictive states in university-affiliated community programs or pure community programs in any year.

The reasons for these findings are difficult to elucidate without directly surveying all ID fellowship applicants and fellows. Applicants targeting academic programs and those targeting other programs may have different educational backgrounds, life experiences, or other priorities when selecting where to apply, influencing how much weight they place on state restrictions when making rank list decisions. Other factors influencing where applicants choose to train may include familial ties or financial limitations that supersede state political climate.

Regardless, one cannot ignore that academic programs in restrictive states suffer from lower match rates than those in permissive states to a significant degree. Because academic programs represent approximately 70% of all adult ID fellowship programs as of 2025 and more than half of ID fellows ultimately continue to practice in the same state in which they train, this match rate differential has implications on the field of ID and access to care [3]. States with restrictions on reproductive and LGBT rights tend to be in regions of the country where access to ID care is already limited.

Geographic disparities in care can be demonstrated in the distribution of new diagnoses of human immunodeficiency virus (HIV) in the United States. In 2022, 52% of new HIV diagnoses were in the Deep South, and the next highest incidence regions were the Southwest, parts of Appalachia, and the eastern half of the Midwest [28]. Aside from a few high-population cities within these regions, rates of HIV pre-exposure prophylaxis treatment were also low, despite the high incidence of HIV [29]. The future of the ID workforce in restrictive states that can help address such disparities is in the students and residents who will train as fellows in restrictive states. Ensuring that match rates of ID fellowships in restrictive states reach parity with those in permissive localities may be imperative to addressing the ID healthcare disparities in restrictive states.

Fellowship programs do not have the power to influence state political climate. However, they do have the power to ensure that they foster a supportive environment to mitigate the potential effects of restrictive political climate on their fellows. Previous studies have shown that people of racial, gender, or sexual minorities often self-select into work environments where there are visible employees sharing their identities or evidence of a supportive work environment [30, 31]. Thus, a strategy may be to establish a diversity, equity, and inclusion (DEI) group at the fellowship institution, such as a female physician or LGBT physician group, to provide support during training. Another strategy is to link fellowship applicants to faculty who also identify as sexual or gender minorities and can speak to their experiences living and practicing in a particular state. There is no single method to improve fellowship match rates in restrictive localities or across the board, but these are possibilities.

A limitation to the current study is that state legislation is fluid, and the status of reproductive and LGBT restrictions can change over time. This was notable in 3 states (Arizona, Missouri, and Ohio) that quickly enacted abortion restrictions through trigger laws or special legislative sessions in 2022 but then had these restrictions later overturned through ballot initiatives [6]. These states were still categorized as having reproductive restrictions for 2 reasons. Politicians in the majority party of some restrictive states have indicated desire to subvert or reverse ballot initiatives that reestablished the right to abortion [32]. Furthermore, before 2022, these states had already moved to restrict abortion, despite the protections set by *Roe v Wade* (1973) [33]. Another limitation to the current study is that changes in fellowship match rates may not necessarily reflect changes in the total number of applications each program receives. The numbers of applications to each program may differ between programs in restrictive and permissive states, but data to perform this analysis are not made publicly available by the NRMP.

In future years, the landscape of reproductive and LGBT rights may continue to evolve as legislation changes. Future studies should aim to create a more refined categorization system that is dynamic alongside changes in state legislation. One potential future study may assess whether match rates in restrictive and permissive states differ among US and international medical graduates. Another potential future study may assess whether applicants who identify as female or LGBT have different application and match rates to programs in restrictive versus permissive states.

In conclusion, the field of ID consistently faces difficulty in filling fellowship positions. Reasons are multifactorial, but state political climate may be a factor. The medical students and residents who represent future ID fellows are rapidly diversifying in a nation where state political climate is fracturing among political lines. As of 2025, many state governments have acted to further tighten existing restrictions on reproductive and LGBT rights, and the US federal government has moved to punish states and institutions that instead protect these rights. These actions present ongoing challenges for future ID physicians deciding where to train and practice. The field of ID cannot ignore that match rates for academic fellowship programs have been significantly lower in programs in states that restrict reproductive and LGBT rights. The findings of our study may guide ID fellowship programs in their efforts to recruit diverse fellows who have the agency to pick where they train.

## Supplementary Material

ofaf534_Supplementary_Data

## Supplementary Data

Supplementary materials are available at *Open Forum Infectious Diseases* online. Consisting of data provided by the authors to benefit the reader, the posted materials are not copyedited and are the sole responsibility of the authors, so questions or comments should be addressed to the corresponding author.
