# Supplementary material for: Association of State Legislation Restricting Reproductive and LGBT Rights With Infectious Diseases Fellowship Match Rates
Source: Open Forum Infect Dis. 2025 Sep 23;12(9):ofaf534. doi: 10.1093/ofid/ofaf534 (PMC12454930; doi:10.1093/ofid/ofaf534)
Supplement: ofaf534_Supplementary_Data [file ofaf534_supplementary_data.docx]

**Supplementary Table 1.** Match Rates of Programs in Permissive and Restrictive States

| **Year** | **Programs in Permissive States** | | | **Programs in Restrictive States** | | | **p (χ2)** |
| --- | --- | --- | --- | --- | --- | --- | --- |
|  | **Matched Positions** | **Unmatched Positions** | **Match Rate (%)** | **Matched Positions** | **Unmatched Positions** | **Match Rate (%)** |  |
| 2017 | 208 | 48 | 81.25 | 100 | 32 | 75.76 | 0.205 |
| 2018 | 211 | 38 | 84.74 | 104 | 35 | 74.82 | 0.017 |
| 2019 | 211 | 41 | 83.73 | 115 | 31 | 78.77 | 0.215 |
| 2020 | 207 | 47 | 81.50 | 114 | 34 | 77.03 | 0.281 |
| 2021 | 239 | 28 | 89.51 | 125 | 25 | 83.33 | 0.069 |
| 2022 | 231 | 42 | 84.62 | 126 | 36 | 77.78 | 0.072 |
| 2023 | 216 | 62 | 77.70 | 111 | 51 | 68.52 | 0.034 |
| 2024 | 194 | 91 | 68.07 | 108 | 55 | 66.26 | 0.694 |
| 2025 | 203 | 76 | 72.76 | 109 | 60 | 64.50 | 0.065 |

*Green highlight indicates statistical significance to p<0.05

**Supplementary Table 2.** Match Rates of Academic Programs and University-Affiliated Community Programs

| **Year** | **All Academic Programs** | | | **All University-Affiliated Community Programs** | | | **p (χ2)** |
| --- | --- | --- | --- | --- | --- | --- | --- |
|  | **Matched Positions** | **Unmatched Positions** | **Match Rate (%)** | **Matched Positions** | **Unmatched Positions** | **Match Rate (%)** |  |
| 2017 | 268 | 52 | 83.75 | 38 | 26 | 59.38 | 0.000 |
| 2018 | 271 | 51 | 84.16 | 43 | 18 | 70.49 | 0.013 |
| 2019 | 274 | 51 | 84.31 | 50 | 17 | 74.63 | 0.057 |
| 2020 | 274 | 53 | 83.79 | 45 | 22 | 67.16 | 0.002 |
| 2021 | 307 | 26 | 92.19 | 52 | 22 | 70.27 | 0.000 |
| 2022 | 295 | 47 | 86.26 | 56 | 24 | 70.00 | 0.000 |
| 2023 | 273 | 77 | 78.00 | 49 | 26 | 65.33 | 0.020 |
| 2024 | 262 | 87 | 75.07 | 46 | 48 | 48.94 | 0.000 |
| 2025 | 252 | 93 | 73.04 | 56 | 27 | 67.47 | 0.310 |

*Green highlight indicates statistical significance to p<0.05

**Supplementary Table 3.** Match Rates of Academic Programs in Permissive and Restrictive States

| **Year** | **Academic Programs in Permissive States** | | | **Academic Programs in Restrictive States** | | | **p (χ2)** |
| --- | --- | --- | --- | --- | --- | --- | --- |
|  | **Matched Positions** | **Unmatched Positions** | **Match Rate (%)** | **Matched Positions** | **Unmatched Positions** | **Match Rate (%)** |  |
| 2017 | 181 | 25 | 87.86 | 87 | 27 | 76.32 | 0.007 |
| 2018 | 180 | 23 | 88.67 | 91 | 28 | 76.47 | 0.004 |
| 2019 | 175 | 28 | 86.21 | 99 | 23 | 81.15 | 0.225 |
| 2020 | 177 | 27 | 86.76 | 97 | 26 | 78.86 | 0.060 |
| 2021 | 199 | 10 | 95.22 | 108 | 16 | 87.10 | 0.008 |
| 2022 | 193 | 22 | 89.77 | 102 | 25 | 80.31 | 0.014 |
| 2023 | 183 | 37 | 83.18 | 90 | 40 | 69.23 | 0.002 |
| 2024 | 172 | 50 | 77.48 | 90 | 37 | 70.87 | 0.170 |
| 2025 | 167 | 48 | 77.67 | 85 | 45 | 65.38 | 0.013 |

*Green highlight indicates statistical significance to p<0.05

**Supplementary Table 4.** Match Rates of University-Affiliated Community Programs in Permissive and Restrictive States

| **Year** | **University-Affiliated Community Programs in Permissive States** | | | **University-Affiliated Community Programs in Restrictive States** | | | **p (χ2)** |
| --- | --- | --- | --- | --- | --- | --- | --- |
|  | **Matched Positions** | **Unmatched Positions** | **Match Rate (%)** | **Matched Positions** | **Unmatched Positions** | **Match Rate (%)** |  |
| 2017 | 26 | 21 | 55.32 | 12 | 5 | 70.59 | 0.272 |
| 2018 | 31 | 13 | 70.45 | 12 | 5 | 70.59 | 0.992 |
| 2019 | 36 | 10 | 78.26 | 14 | 7 | 66.67 | 0.312 |
| 2020 | 30 | 17 | 63.83 | 15 | 5 | 75.00 | 0.373 |
| 2021 | 38 | 15 | 71.70 | 14 | 7 | 66.67 | 0.669 |
| 2022 | 35 | 18 | 66.04 | 21 | 6 | 77.78 | 0.279 |
| 2023 | 32 | 18 | 64.00 | 17 | 8 | 68.00 | 0.731 |
| 2024 | 22 | 32 | 40.74 | 14 | 16 | 46.67 | 0.599 |
| 2025 | 34 | 21 | 61.82 | 22 | 6 | 78.57 | 0.123 |

*Green highlight indicates statistical significance to p<0.05

**Supplementary Table 5.** Match Rates of Pure Community Programs in Permissive and Restrictive States

| **Year** | **Pure Community Programs in Permissive States** | | | **Pure Community Programs in Restrictive States** | | | **p (χ2)** |
| --- | --- | --- | --- | --- | --- | --- | --- |
|  | **Matched Positions** | **Unmatched Positions** | **Match Rate (%)** | **Matched Positions** | **Unmatched Positions** | **Match Rate (%)** |  |
| 2017 | 1 | 2 | 33.33 | 1 | 0 | 100.00 | 0.414 |
| 2018 | 0 | 2 | 0.00 | 1 | 2 | 33.33 | 0.683 |
| 2019 | 0 | 3 | 0.00 | 2 | 1 | 66.67 | 0.317 |
| 2020 | 0 | 3 | 0.00 | 2 | 3 | 40.00 | 0.527 |
| 2021 | 2 | 3 | 40.00 | 3 | 2 | 60.00 | 0.655 |
| 2022 | 3 | 2 | 60.00 | 3 | 5 | 37.50 | 0.591 |
| 2023 | 1 | 7 | 12.50 | 4 | 3 | 57.14 | 0.291 |
| 2024 | 0 | 9 | 0.00 | 4 | 2 | 66.67 | 0.140 |
| 2025 | 2 | 7 | 22.22 | 2 | 9 | 18.18 | 0.920 |

*Green highlight indicates statistical significance to p<0.05

**Supplementary Table 6.** Match Rates of Academic Programs and Pure Community Programs

| **Year** | **All Academic Programs** | | | **All Pure Community Programs** | | | **p (χ2)** |
| --- | --- | --- | --- | --- | --- | --- | --- |
|  | **Matched Positions** | **Unmatched Positions** | **Match Rate (%)** | **Matched Positions** | **Unmatched Positions** | **Match Rate (%)** |  |
| 2017 | 268 | 52 | 83.75 | 2 | 2 | 50.00 | 0.072 |
| 2018 | 271 | 51 | 84.16 | 1 | 4 | 20.00 | 0.000 |
| 2019 | 274 | 51 | 84.31 | 2 | 4 | 33.33 | 0.001 |
| 2020 | 274 | 53 | 83.79 | 2 | 6 | 25.00 | 0.000 |
| 2021 | 307 | 26 | 92.19 | 5 | 5 | 50.00 | 0.000 |
| 2022 | 295 | 47 | 86.26 | 6 | 7 | 46.15 | 0.000 |
| 2023 | 273 | 77 | 78.00 | 5 | 10 | 33.33 | 0.000 |
| 2024 | 262 | 87 | 75.07 | 4 | 11 | 26.67 | 0.000 |
| 2025 | 252 | 93 | 73.04 | 4 | 16 | 20.00 | 0.000 |

*Green highlight indicates statistical significance to p<0.05

**Supplementary Table 7.** Match Rates of University-Affiliated Community Programs and Pure Community Programs

| **Year** | **All University-Affiliated Community Programs** | | | **All Pure Community Programs** | | | **p (χ2)** |
| --- | --- | --- | --- | --- | --- | --- | --- |
|  | **Matched Positions** | **Unmatched Positions** | **Match Rate (%)** | **Matched Positions** | **Unmatched Positions** | **Match Rate (%)** |  |
| 2017 | 38 | 26 | 59.38 | 2 | 2 | 50.00 | 0.712 |
| 2018 | 43 | 18 | 70.49 | 1 | 4 | 20.00 | 0.021 |
| 2019 | 50 | 17 | 74.63 | 2 | 4 | 33.33 | 0.032 |
| 2020 | 45 | 22 | 67.16 | 2 | 6 | 25.00 | 0.020 |
| 2021 | 52 | 22 | 70.27 | 5 | 5 | 50.00 | 0.198 |
| 2022 | 56 | 24 | 70.00 | 6 | 7 | 46.15 | 0.091 |
| 2023 | 49 | 26 | 65.33 | 5 | 10 | 33.33 | 0.021 |
| 2024 | 46 | 48 | 48.94 | 4 | 11 | 26.67 | 0.239 |
| 2025 | 56 | 27 | 67.47 | 4 | 16 | 20.00 | 0.000 |

*Green highlight indicates statistical significance to p<0.05
